# Group I introns and associated homing endonuclease genes reveals a clinal structure for *Porphyra spiralis *var. *amplifolia *(Bangiales, Rhodophyta) along the Eastern coast of South America

**DOI:** 10.1186/1471-2148-8-308

**Published:** 2008-11-07

**Authors:** Daniela Milstein, Mariana C Oliveira, Felipe M Martins, Sergio R Matioli

**Affiliations:** 1 Departamento de Botânica, Instituto de Biociências, Universidade de São Paulo, Rua do Matão, 277, cep 05508-900, Brazil; 2 Departamento de Genética e Biologia Evolutiva, Instituto de Biociências, Universidade de São Paulo, Rua do Matão, 277, cep 05508-900, Brazil

## Abstract

**Background:**

Group I introns are found in the nuclear small subunit ribosomal RNA gene (SSU rDNA) of some species of the genus *Porphyra *(Bangiales, Rhodophyta). Size polymorphisms in group I introns has been interpreted as the result of the degeneration of homing endonuclease genes (HEG) inserted in peripheral loops of intron paired elements. In this study, intron size polymorphisms were characterized for different *Porphyra spiralis *var. *amplifolia *(*PSA*) populations on the Southern Brazilian coast, and were used to infer genetic relationships and genetic structure of these *PSA *populations, in addition to *cox*2-3 and *rbc*L-S regions. Introns of different sizes were tested qualitatively for *in vitro *self-splicing.

**Results:**

Five intron size polymorphisms within 17 haplotypes were obtained from 80 individuals representing eight localities along the distribution of *PSA *in the Eastern coast of South America. In order to infer genetic structure and genetic relationships of *PSA*, these polymorphisms and haplotypes were used as markers for pairwise Fst analyses, Mantel's test and median joining network. The five *cox*2-3 haplotypes and the unique *rbc*L-S haplotype were used as markers for summary statistics, neutrality tests Tajima's *D *and Fu's *Fs *and for median joining network analyses. An event of demographic expansion from a population with low effective number, followed by a pattern of isolation by distance was obtained for *PSA *populations with the three analyses. *In vitro *experiments have shown that introns of different lengths were able to self-splice from pre-RNA transcripts.

**Conclusion:**

The findings indicated that degenerated HEGs are reminiscent of the presence of a full-length and functional HEG, once fixed for *PSA *populations. The cline of HEG degeneration determined the pattern of isolation by distance. Analyses with the other markers indicated an event of demographic expansion from a population with low effective number. The different degrees of degeneration of the HEG do not refrain intron self-splicing. To our knowledge, this was the first study to address intraspecific evolutionary history of a nuclear group I intron; to use nuclear, mitochondrial and chloroplast DNA for population level analyses of *Porphyra; *and intron size polymorphism as a marker for population genetics.

## Background

Group I introns belong to a family of RNAs with catalytic activities. These ribozymes are mobile elements inserted within coding sequences of nuclear rDNA, chloroplast and mitochondrial genomes of some eukaryotes; and less frequently within coding sequences of eubacteria, phages and viruses. Group I introns catalyze their own excision (self-splicing) from pre-mRNA when mature RNA is being processed. The exact site of intron excision and the perfect reestablishment of the interrupted message are defined by specific interactions between intron and exons, determined by a conserved secondary structure. Group I introns fold on a structure, forming 10 conserved paired elements (P1-P10) with a conserved catalytic core [reviewed in [1]].

Size polymorphisms in group I introns have been described [2-5] and are occasionally generated by the insertion of a mobile element such as homing endonuclease genes (HEG) in peripheral loops of intron paired elements P1, P2, P6, P8 and P9 [4,6]. Homing endonuclease genes encode for site specific homing endonucleases (HEs), which in genetic crosses between an HEG containing intron allele and an intronless allele, recognize the intron insertion site and catalyze a double strand break. The intronless allele is then repaired using the HEG containing intron allele as a template. This mechanism of intron mobility is known as homing [6]. Homing endonucleases are classified in five different families according to conserved protein motifs and functional and structural properties: LAGLIDADG; GIY-YIG; H-N-H; His-Cys box [6-8]; and the recently described PD-(D/E)-XK motif [9]. His-Cys box motifs are identified in HE exclusively associated to nuclear group I introns [10]. Homing endonucleases were described for fungi, protists, bacteria and viruses, but with unknown function for the hosts [6].

Descriptions of group I introns in Rhodophyta are limited to a few genera [11-13], although it is commonly reported for the genera *Porphyra *and *Bangia *(Bangiales, Rhodophyta) [13-15]. A survey for group I introns presence in the order (Bangiales) described by Müller *et al*. [5], indicated that this order is a particularly rich in these introns. The use of introns as molecular markers at the intra-specific level is very limited [2,16,17]. Usually in Rhodophyta, analyses at the intra-specific level are addressed with molecular markers such as the nuclear rDNA internal transcribed spacer (ITS) 1 and 2 [18,19]; the plastidial spacer between the ribulose-1, 5-bisphosphate carboxylase-oxygenase large subunit (*rbc*L) and the small subunit (*rbc*S) genes (*rbc*L-S) [20,21], and the mitochondrial spacer between the cytochrome oxidase subunit 2 and subunit 3 genes (*cox*2-3) [19,21-23].

In a previous work, Oliveira and Ragan [2] characterized introns of different sizes inserted in the nuclear small subunit rRNA gene (SSU rDNA) close to the 3' end (intron S1506) of three *Porphyra spiralis *var. *amplifolia *(*PSA*) individuals collected at different sites on the Southern Brazilian coast. Open reading frames (ORFs) with His-Cys Box motifs were described inserted in the P1 paired element, confined within the conserved pair U*G, located in the SSU rDNA exon and in the intron respectively in the complementary strand. This region is known as P1-extension [4]. These findings prompted us to: 1) characterize introns size polymorphisms at different *PSA *populations on the Eastern coast of South America; 2) Infer genetic relationships and population structure of *PSA *populations using introns in addition to *rbc*L-S and *cox*2-3 regions as genetic markers; and 3) Verify if the different polymorphisms in peripheral loop of intron P1 paired element affected qualitatively introns excision, through an *in vitro *self-splicing assay.

## Methods

### DNA extraction, PCR amplification and sequencing

**Figure 1:**
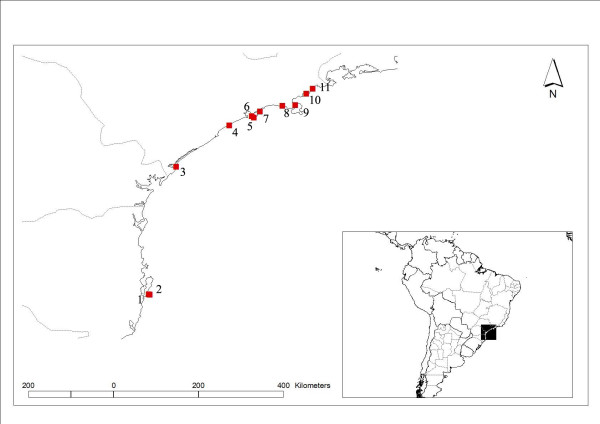
**Map of South America highlighting *Porphyra spiralis *var. *amplifolia *(*PSA*) collection sites**. 1- *PSA*-A; 2- *PSA*-L; 3- *PSA-*R*; 4- *PSA*-I; 5- *PSA*-S; 6- *PSA*-T; 7- *PSA*-G; 8- *PSA*-C; 9- *PSA*-D*; 10- *PSA*-B*; 11- *PSA*-V. Additional information on collections sites are presented on Table 1. * Data obtained from Oliveira and Ragan [2].

Population samples of *Porphyra spiralis *var. *amplifolia *were collected at eight different sites in the Southern Brazilian shore (Table 1, Figure 1). A minimum of 10 individuals were obtained from each site. Gametophyte blades were identified based on morphological description [24], and did not present any meaningful morphological variation among and within populations. Samples were screened for epiphytes using a stereomicroscope, and stored individually in silica gel. Each individual was ground in liquid nitrogen and total genomic DNA was extracted using the "DNeasy Plant Mini Kit" (Qiagen, Santa Clarita, CA), according to manufacturer's specifications. Voucher specimens are deposited at University of São Paulo herbarium (SPF, Table 1).

**Table 1:** *Porphyra spiralis *var. *amplifolia *(*PSA*) collection information.

Population	Collection location	Latitude (S)/Longitude (W)	Collector	Date	Voucher
*PSA*-V	Vermelha do Norte, Ubatuba, SP	23°25'16.96"/45°02'21.75"	MCO	Aug 1998	SPF 56191
*PSA*-B^1^	Fortaleza, Ubatuba, SP	23°31'55.48"/45°09'42.28"	MCO	Oct 1990	*
*PSA*-D^1^	Cebimar, São Sebastião, SP	23°49'41.70"/45°25'20.87"	MCO	Oct 1990	*
*PSA*-C	Baleia, São Sebastião, SP	23°46'49.78"/45°39'51.53"	DM	Aug 2002	SPF 56183
*PSA*-G	Tijucopava, Guarujá, SP	23°54'54.72"/46°10'03.21"	DM	Jul 2002	SPF 56185
*PSA*-S	Itaipu, Santos, SP	24°01'12.02"/46°23'57.57"	ECO	Aug 1997	SPF 56189
*PSA*-T	Tombo, Guarujá, SP	24°00'45.84"/46°16'08.38"	DM	Jul 2002	SPF 56180
*PSA*-I	Cibratel, Itanhaém, SP	24°11'26.90"/46°47'33.16"	MCO	Aug 1997	SPF 56186
*PSA*-R^1^	Ilha do Cardoso, SP	25°06'29.49"/47°53'41.20"	EJP	Aug 1989	*
*PSA*-A	Ponta da Armação, Florianópolis, SC	27°44'50.77"/48°29'54.22"	ECO	Nov 1997	SPF 56181
*PSA*-L	Lagoinha, Florianópolis, SC	27°46'43.79"/48°29'15.84"	ECO	Nov 1997	SPF 56187

SC- Santa Catarina State, SP- São Paulo State. Collectors: DM- Daniela Milstein, EJP – Edison José de Paula, ECO – Eurico Cabral de Oliveira, MCO- Mariana Cabral de Oliveira. * Conchocelis phase in culture (Laboratório de Algas Marinhas Édison J. de Paula, Instituto de Biociências, USP). ^1 ^Data obtained from Oliveira and Ragan [2].

Total DNA was extracted from 10 individuals for each of the eight geographic locations. Primers 1400F and 18S3' were used to amplify part of the 3' end of the first SSU rDNA exon + intron + the 5' end of the second SSU rDNA exon; as a positive control for intron presence in the multiple SSU rDNA copies [25] primers 1400F and iR2 were used to amplify HEG-containing ORF, including the flanking 213 bp of the SSU rDNA 5'exon and 175 bp of the intron. Primers COX 2F and COX 3R were used to amplify the 3' end of *cox*2 gene + spacer + 5' end of *cox*3 gene; and primers F993 and RBCS3'R were used to amplify the 3' end of *rbc*L gene + spacer + 5' end of *rbc*S gene.

PCR amplification conditions for a total volume of 50 μL were: 1× PCR buffer, 1.5 mM MgCl_2_, 0.2 mM each dNTP, 0.2 μM each primer, 20 ng of genomic DNA and 1.25 U of *Taq *DNA polymerase (Promega Corporation, Madison, WI). All PCR reactions were performed in a MiniCycler thermocycler (MJ Research, Watertown, MA) and cycles varied according to the region to be amplified: Introns – 94°C for 4 min, 35 cycles of 94°C for 30 sec, 60°C for 1 min, 72°C for 2 min, and a final extension step at 72°C for 7 min. *cox*2-3 region – 94°C for 4 min, 5 cycles of 93°C for 1 min, 45°C for 1 min, 72°C for 1 min, followed by 30 cycles of 93°C for 30 sec, 55°C for 30 sec, 72°C for 30 sec, and a final extension step at 72°C for 5 min. *rbc*L-S region – 94°C for 4 min, 35 cycles of 94°C for 1 min, 42°C for 1 min, 72°C for 1 min and 30 sec, and a final extension step at 72°C for 10 min. Primers used for amplification and sequencing are listed in Table 2. Negative controls for PCR reactions, that included all reagents except DNA template, were performed. At least three independent PCR reactions were pooled together before sequencing [26].

**Table 2:** Primers used for intron amplification and sequencing.

Primers	Sequences	Position in *PSA*	References
1400F	5'-TGTACACACCGCCCGTC-3'	SSU rDNA, 1659	Oliveira and Ragan [2]
iF-1	5'-ACAAGGTTTCCGAAAGGG-3'	Intron, 1798	Oliveira and Ragan [2]
iR-3	5'-TTAATGTCGTGACCGCGCA-3'	Intron, 2306	Oliveira and Ragan [2]
iF-2	5'-AAGTCGCTTTTGTTGGC-3'	Intron, 2382	Oliveira and Ragan [2]
iR-2	5'-TTCGGACTGACTGCGTCG-3'	Intron, 2461	Oliveira and Ragan [2]
iF-3	5'-CGCTGGATGGTAATAAGGTG-3'	Intron, 2580	Oliveira and Ragan [2]
iR-1	5'-GACTCTGCTTTCGCAG-3'	Intron, 2730	Oliveira and Ragan [2]
18S3'	5'-GATCCTTCTGCAGGTTCACCTACGGAA-3'	SSU rDNA, 2860	Oliveira and Ragan [2]
COX 2F	5'- GTACCWTCTTTDRGRRKDAAATGTGATGC -3'	*cox*2-3, 1	Zuccarello *et al*. [43]
COX 3R	5'- GGATCTACWAGATGRAAWGGATGTC -3'	*cox*2-3, 433	Zuccarello *et al*. [43]
F993	5'- GGTACTGTTGTAGGTAAATTWGAAGG -3'	*rbc*L - S, 1	Freshwater *et al*. [46]
RBCS3'R	5'- GTTCTTGTGTTAATCTCAC -3'	*rbc*L - S, 571	Freshwater *et al*. [46]

PCR products were purified using the MicroSpin™ S-300 HR Columns (Amersham Pharmacia Biotech, Piscataway, NJ), and were directly sequenced on an ABI PRISM™ 310 Genetic Analyser or 3100 DNA Sequencer (Applied Biosystems, Foster City, CA) using the sequencing kit " BigDye™ Terminator Cycle Sequencing Ready Reaction" (Applied Biosystems) according to manufacturer's specifications. Sequences were manually assembled and aligned with BioEdit version 5.0.6 [27]. Ambiguous nucleotides within the same individual sequence position were checked against the sequencing chromatograms, to confirm validity of the nucleotide.

### Analyses of population structure

Software DNAsp [28] were used to calculate summary statistics (H), neutrality tests Tajima's *D *[29] and Fu's *Fs *[30]. For median joining (MJ) network analyses, sequences previously aligned with Fluxus' DNA Alignment 1.121 software [31] were input in the program NETWORK 4.1 [31]. All parameters implemented in NETWORK were set to default: Characters weights (10 for all characters), transversions/transitions ratios (1:1) and the distance calculation method (connection cost). Parameter epsilon, a weighted genetic distance measure, was set to 0. Population genetics analyses for intron + HEG were carried out using Arlequin [32]. The dataset was input as sequence length polymorphism (based on PCR results) with ten individuals per population. Introns were grouped in four categories according to their size: 1- 616 bp; 2- from 791 to 792 bp; 3- 909 bp; 4- from 1054 to 1058 bp. This dataset was analyzed for F-statistics implementation. This software was also used for Mantel's test of isolation by distance. *PSA*-B, *PSA*-D and *PSA*-R individuals were excluded from these analyses (data available for only one individual per population).

Intron nomenclature adopted in this work (*i*.*e*. S1506) was modified from Johansen and Haugen [33] and the insertion location of the introns is given according to the reference position in *Escherichia coli *SSU rDNA.

### Cloning and *in vitro *transcription

Primers 1400F and 18S3' were used for the amplification of the nuclear SSU rDNA intron, including the flanking 213 bp of the SSU rDNA 5'exon and 27 bp of the SSU rDNA 3'exon. Amplicons of one individual of *PSA*-G, *PSA*-L, *PSA*-T and *PSA*-V populations were cloned according to manufacturer's specifications in pGEM^®^-T vectors (Promega Corporation, Madison, WI), and were replicated in *E. coli *DH10B. Plasmids were recovered and purified with Wizard^® ^plus SV Minipreps DNA Purification System (Promega) according to manufacturer's protocol. Inserts were PCR amplified with primers T7 and 18S3' for an *in vitro *transcription assay (Table 2), and purified with the kit Wizard^® ^SV Gel and PCR Clean up System (Promega) according to manufacturer's protocol. Negative controls for PCR reactions were performed.

Transcription reactions (50 μL) were performed with 1 μg of purified PCR products and T7 RNA polymerase enzyme, in T7 RiboMAX™ Express large scale RNA production system kit (Promega) according to manufacturer's protocol. Transcription reactions were incubated at 45°C for 45 min, then were digested with RQ1 RNase-free DNase (Promega) and RNA transcripts were extracted with phenol (pH 4.7): chlorophorm: isoamyl alcohol (125:24:1).

### Intron *in vitro *self-splicing assays

The extracted RNAs were tested for intron qualitative *in vitro *self-splicing by the following assay: an aliquot of each transcription reaction was incubated at 45°C for 45 min, in the presence of the self-splicing buffer as described in Sogin and Edman [34]: 100 mM (NH_4_)_2 _SO_4_, 50 mM Tris-HCl pH 7.5, 60 mM MgCl_2 _and 0.2 mM GTP. The RNA was extracted with phenol: chlorophorm: isoamyl alcohol (25:24:1). To verify if introns self-spliced from RNA, 2 μL of RNA were denatured at 70°C for 10 min in 18 μL of formamide denaturing buffer (according to manufacturer's protocol) and were visualized in an ethidium bromide stained 2% agarose gel [35].

Reverse transcription reactions followed by PCR (50 μL) were carried out with 1X AccessQuick™ Master Mix buffer (Promega), approximately 10 ng RNA, 0.2 μM of each primer (1400F and 18S3') and 5 units AMV reverse transcriptase (Promega). For cDNA synthesis, the reactions were incubated at 45°C for 45 min followed by PCR cycle: 95°C for 2 min; 40 cycles at 95°C for 30 sec and 60°C for 30 sec; with a final extension step at 72°C for 5 min according to manufacturer's protocol. All PCR reactions included negative controls and were performed in a MiniCycler thermocycler (MJ Research).

PCR products were purified from agarose gels using Wizard^® ^SV Gel and PCR clean up system kit (Promega) and were re-amplified with primers 1400F and 18S3' as described above. The PCR cycle used was as described for RT-PCR. PCR products were purified with Wizard^® ^SV Gel and PCR clean up system kit (Promega) and were directly sequenced as described above.

## Results

### Characterization of introns size polymorphisms at different *PSA *populations

Group I S1506 introns from 80 *Porphyra spiralis *var. *amplifolia *(*PSA*) collected at eight different sites in the Southern Brazilian shore were PCR amplified. Visualization in 0.7% agarose gel unveiled four introns size polymorphism among different *PSA *populations. The smallest introns were amplified from *PSA*-V population (616 bp) and the largest introns were amplified from *PSA*-A, *PSA*-I, *PSA*-L and *PSA*-T populations (1054-8 bp; Table 3). Intron size polymorphisms within a population were detected only for *PSA*-S and *PSA*-T collections. Presence of two introns of different sizes occurring in the same sample was observed for one individual from *PSA*-S (792 bp + 909 bp) and for one individual from *PSA*-T (792 bp + 1055 bp). Based on these PCR results, introns from 10 individuals of *PSA*-S, *PSA*-T and *PSA*-V were sequenced, and for the remaining populations, introns from two individuals per population were sequenced. Sequencing data from individuals *PSA*-B, *PSA*-D and *PSA*-R [2] were obtained from the GenBank. In total, 44 introns sequences were analyzed [GenBank accession numbers from FJ147627 to FJ147667].

**Table 3:** Seventeen haplotypes (H1 to H17) distributed over 11 *Porphyra spiralis *var. *amplifolia *(*PSA*) populations (Table 1).

Haplotype/Intron size	*PSA *Populations	ORF	His-Cys
H1 - 1056 bp	*PSA*-A	137 aa	Yes, f/s
H2 - 1058 bp	*PSA*-I	138 aa	Yes
H3 - 1054 bp	*PSA*-R^1,2^	137 aa	Yes, f/s
H4 - 1055 bp	*PSA*-L	137 aa	Yes
H5 - 1055 bp	*PSA*-I, -L, -T	137 aa	Yes
H6 - 909 bp	*PSA*-D^1^	87 aa	Yes, f/s
H7 - 909 bp	*PSA*-S, -T	88 aa	Yes, f/s
H8 - 791 bp	*PSA*-C	49 aa	No
H9 - 792 bp	*PSA*-G	50 aa	No
H10 - 792 bp	*PSA*-G, -S, -T	50 aa	No
H11 - 792 bp	*PSA*-T	50 aa	No
H12 - 792 bp	*PSA*-S	50 aa	No
H13 - 791 bp	*PSA*-S	50 aa	No
H14 - 744 bp	*PSA*-B^1^	45 aa	No
H15 - 616 bp	*PSA*-V	8 aa	No
H16 - 616 bp	*PSA*-V	8 aa	No
H17 - 616 bp	*PSA*-V	8 aa	No

Intron sizes in base pairs (bp), homing endonuclease open reading frames (ORF) sizes in amino acids (aa) and if the His-Cys box motif is present are indicated; f/s indicates if frame shifts corrections are necessary for establishing the correct His-Cys box reading frame. Data obtained from: ^1 ^Oliveira and Ragan [2], ^2 ^Haugen *et al *[4].

Introns size polymorphisms are due to insertions from 42 to 482 bp in intron P1 paired element. Variability among the 44 *PSA *sequences (introns and P1-extension) yielded 17 different haplotypes (see additional file 1), with haplotype diversity (H) of 0.895. P1-extension is the most variable region presenting nucleotide substitutions and indels. When P1-extension was excluded from this comparison, only six different haplotypes were obtained with H = 0.175, and the substitutions observed were limited to intron unpaired terminal loops (data not shown). The neutrality tests results for introns without the P1-extension were all negative and significant (Tajima's D = -1.99, p < 0.05; Fu's F = -3.63, p < 0.02), suggesting a rapid population expansion.

**Figure 2:**
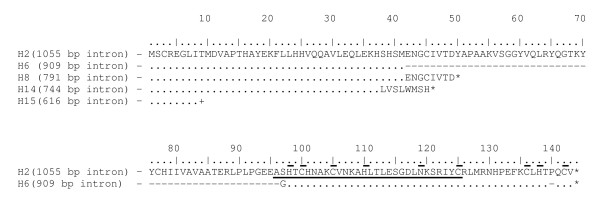
**Translation of homing endonuclease gene open reading frames of the five size polymorphisms**. The His-Cys box motif is underlined and zinc binding sites and active sites found in I-*Ppo*I endonuclease are indicated by line above amino acids. Stop codon is indicated by asterisk. End of sequence without stop codon is indicated by +. H – haplotype.

Complementary strands of P1-extensions of the 17 haplotypes were translated *in silico *to amino acid sequences according to Haugen *et al*. [4]. Open reading frames from eight to 150 amino acids, were generated (Table 3, Figure 2) and were blasted against other available proteins in GenBank with BLASTP [36]. Start codons for HEG were found in all ORFs, however when compared to others HEs, premature stop codons or stop codon deletions were observed. His-Cys box motifs, zinc binding sites and active sites were characterized on intron-coding complementary strands for six *PSA *individuals from three different populations (Haplotypes H2, H4 and H5, Table 3, Figure 2). For the remaining introns, His-Cys box motifs, zinc binding sites and active sites were only verified when frame-shifts corrections were manually inserted *in silico*, or were absent. These results indicate that P1-extension of the 17 haplotypes are degenerated HEG.

### *Cox*2-3 and *rbc*L-S analyses

*Cox*2-3 region was PCR amplified and sequenced for five individuals of each population analyzed in this work [GenBank accession numbers from FJ147587 to FJ147626]. The amplified region has a total length of 457 bp for all individuals, being the *cox*2-3 spacer 167 bp long. Variability among the 40 *PSA cox*2-3 region sequences analyzed, yielded five different haplotypes (Table 4). Haplotypes differed from each other by one or two base-pairs (0.2% to 0.7%), with H = 0.426. Similar to the results obtained for the intron analyses, the Tajima's D values for *cox*2-3 were negative (-1.42), but only marginally significant (0.05 < p < 0.10).

**Table 4:** *Cox*2-3 region haplotype frequencies for each population of *Porphyra spiralis *var. *amplifolia *(*PSA*).

*PSA *Population	Hapl A	Hapl B	Hapl C	Hapl D	Hapl E
*PSA*-A	5	-	-	-	-
*PSA*-C	5	-	-	-	-
*PSA*-G	5	-	-	-	-
*PSA*-I	4	-	-	-	1
*PSA*-L	-	-	5	-	-
*PSA*-S	4	-	-	1	-
*PSA*-T	5	-	-	-	-
*PSA*-V	2	3	-	-	-
Total	30	3	5	1	1

*RbcL*-S region was PCR amplified and sequenced for five individuals of populations *PSA*-S, *PSA*-T and *PSA*-V, and for two individuals of the remaining populations. The region amplified is 570 bp long for all individuals, being the *rbc*L-S spacer 77 bp long. A single haplotype was obtained for all 25 analyzed individuals, with no divergence within and among populations [GenBank accession numbers from FJ147668 to FJ147694].

### Genetic relationships and population structure of *PSA *populations

**Figure 3:**
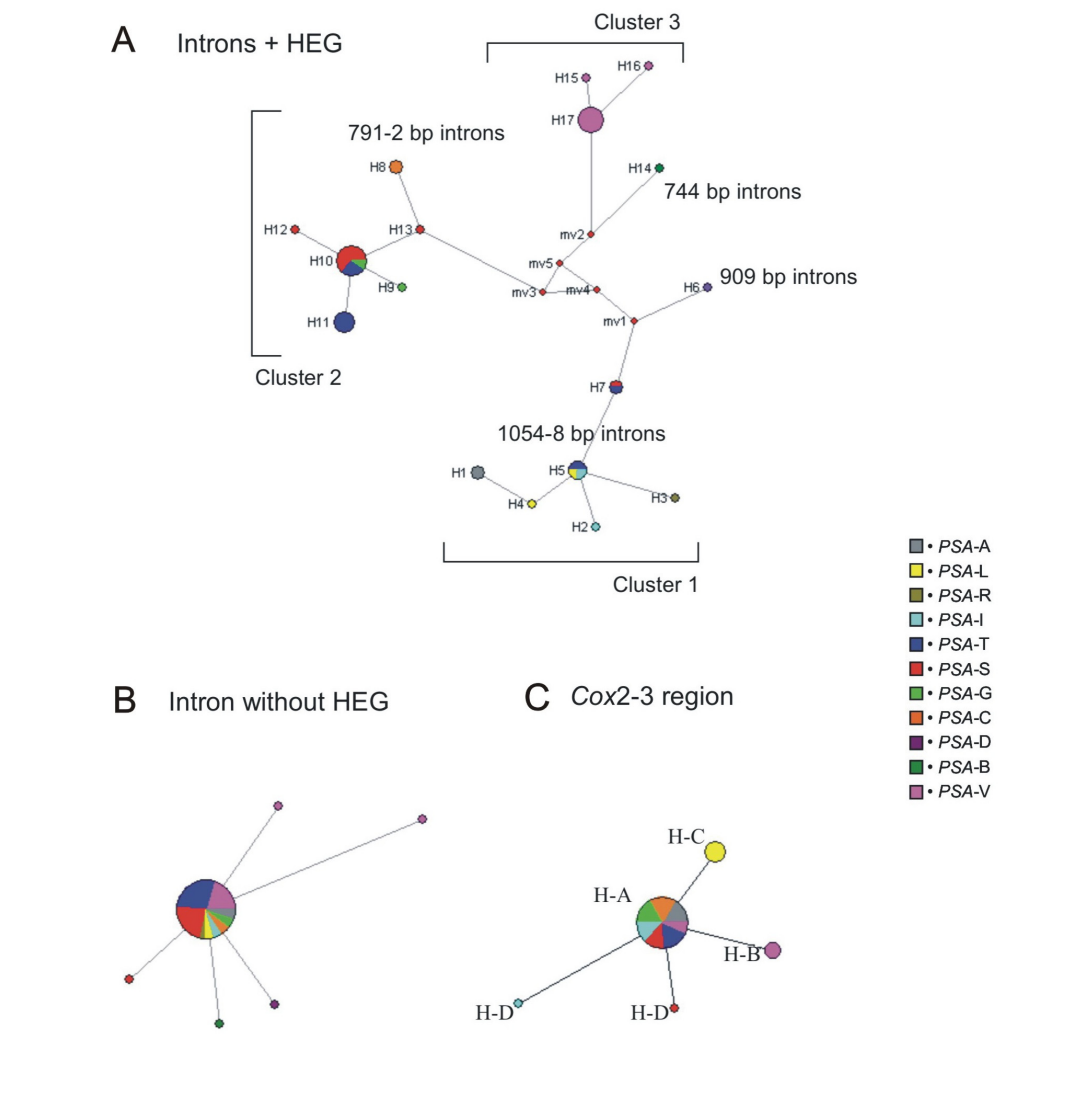
**Median-joining networks: A – Introns + homing endonuclease gene (HEG); B – and introns without HEG; C – *Cox*2-3 region**. Circles indicate sequence types and circle sizes are proportional to haplotype frequency. Lines indicate substitutions (not on scale). MV- median vector, H- haplotype. Haplotypes were not plotted on intron without HEG network.

To determine the genetic relationships among the studied *PSA *populations, three median joining networks were constructed. The first one included intron + HEG sequences (Figure 3A), the second was performed with intron without HEG sequence data (Figure 3B), and the third was constructed with *cox*2-3 region sequences (Figure 3C).

The network generated for intron + HEG sequences shows three main clusters connected by median vectors, which represent missing intermediates, that is extant haplotype that was not sampled or an extinct ancestral haplotype [37]. The first cluster comprises introns with 1054-8 bp represented by haplotypes H1 to H5 and introns with 909 bp represented by haplotypes H6 and H7. The most common haplotype in this cluster is H5, which occurs in three geographically distant populations (*PSA*-L, *PSA*-I, *PSA*-T). Haplotypes H2, H4 and H5 present the intact His-Cys box motif whereas haplotypes H1 and H3 present His-Cys box only when frame shifts are inserted. Haplotypes H6 and H7 present extensive deletions in the HEG, but still have part of the His-Cys box motif. The second cluster comprises exclusively 791-2 bp introns without His-Cys box motif, represented by haplotypes H8 to H13. The most common haplotype in this cluster is H10, which is found in populations *PSA*-G, *PSA*- S and *PSA*-T. The third cluster comprises the smaller introns (616 bp and 744 bp) represented by haplotypes H14 to H17. The most common haplotype in this cluster is H17, which is from population *PSA*-V. The intron + HEG network shows a pattern of isolation by distance for *PSA *populations.

The results for pairwise Fst analyses of frequency of allele size polymorphisms are presented in Table 5. It was possible to note three distinct patterns of significant Fst values, according to population geographic distribution. Populations *PSA*-A,*PSA*-I and *PSA*-L presented significant Fst values when compared to populations *PSA*-C, *PSA*-G, *PSA*-S and *PSA*-T, and all of them presented significant Fst values when compared to population *PSA*-V. Mantel's test results found significant correlation between Fst values and geographic distance (p = 0.028) corroborating the hypothesis of isolation by distance suggested by intron + HEG network.

**Table 5:** Population Pairwise Fst.

Population	*PSA*-A	*PSA*-C	*PSA*-G	*PSA*-I	*PSA*-L	*PSA*-S	*PSA*-T	*PSA*-V
*PSA*-A		+	+	-	-	+	+	+
*PSA*-C	1.0000		-	+	+	-	-	+
*PSA*-G	1.0000	0.0000		+	+	-	-	+
*PSA*-I	0.0000	1.0000	1.0000		-	+	+	+
*PSA*-L	0.0000	1.0000	1.0000	0.0000		+	+	+
*PSA*-S	0.9000	0.000	0.000	0.9000	0.9000		-	+
*PSA*-T	0.6996	0.1205	0.1205	0.6996	0.6996	-0.004		+
*PSA*-V	1.0000	1.0000	1.0000	1.0000	1.0000	0.9000	0.7537	

Pairwise Fst values of intron size polymorphisms of *Porphyra spiralis *var. *amplifolia *populations (*PSA*, lower left). Significant values are represented by the plus sign, and no significant values are represented by the minus sign (upper right).

The minimum spanning tree generated for intron without HEG (Figure 3B) and *cox*2-3 region sequences (Figure 3C) exhibit a star-like topology. For intron without HEG, the most frequent central haplotype occurred in 9 out of 11 collection sites (39 of the 44 individuals; 89%). For *cox*2-3 region sequences, the majority of the individuals (7 of the 8 populations accounting for 30 out of 40 individuals analyzed; 75%) possess the most frequent central haplotype. According to both networks topologies, a recent population expansion was detected for these markers.

### Self splicing assays

Introns and exons flanking regions of one individual from populations *PSA*-V (616 bp), *PSA*-G (792 bp), *PSA*-T (909 bp) and *PSA*-L (1055 bp) were cloned for *in vitro *transcription. Pre-RNA of the four individuals were incubated at 45°C for 45 min to verify the self-splicing reaction. The self-splicing reaction was observed, however just a part of the introns was spliced, while the other part remained attached to exons (as pre-RNA, additional file 2).

To confirm whether the exons were ligated, a RT-PCR reaction was performed with primers 1400F and 18S3' using the RNA previously incubated in the conditions described above. The bands were excised from the gel and re-amplified with primers 1400F and 18S3' which anchors in the exons. The results of the re-amplification are shown in additional file 3. Amplicons were sequenced and the smaller bands were the joined 5' and 3' exons, presenting the reconstructed insertion site.

## Discussion

### Characterization of introns size polymorphisms at different *PSA *populations

Group I introns are well documented in the literature occurring in the red algal genera *Bangia *and *Porphyra *(Order Bangiales) [5,13-15]. Some of these group I introns present ORFs of different sizes inserted in their P1 and P2 paired elements [3-5]. Size variation in these ORFs represents different stages of the HEG cycle (full length, degenerated or absent). Goddard and Burt [38] postulated that the HE coded by an intron recognizes the intron insertion site in an intronless population, invade it by lateral transfer and then it is vertically transmitted to the offspring. After being fixed with high frequencies in a population, the HEG degenerates to a non-functional state, and then the intron and the HEG tend to be lost. In this way, the intron recognition site is reestablished becoming available to be invaded again by an active HEG-containing intron from the same species or from a closely related species, thus restarting the homing cycle.

In a previous work, Oliveira and Ragan [2] characterized three size polymorphisms for group I introns from three *PSA *individuals. In this work, two more sizes were characterized, in a total of five different sizes distributed in 11 *PSA *populations along the Brazilian coast. According to the cycle proposed by Goddard and Burt [38], HEGs can be found in three different character states: functional (full length), nonfunctional (degenerated) and absent (both HEG and intron). In *PSA *populations analyzed in this work, we could only detect the nonfunctional state represented by HEG degeneration, indicating that full length HEG containing intron was once fixed for these populations. The different states of the intron + HEG are not always found within natural populations, probably as a result of insufficient sampling [1].

Müller *et al*. [5] evaluated if the cycle proposed by Goddard and Burt [38], applied to group I introns present in the order Bangiales. Presence of introns containing degenerated HEGs, presence of introns without HEG and absence of introns, all these states scattered along individuals from different species, indicated that Goddard & Burt [38] model is supported by intron + HEG distribution in the order Bangiales.

Of the 44 introns sequences, only six presented the intact His-Cys box motif. Although these ORFs did not present frameshifts mutation, they terminated prematurely relatively to the amino acid sequence for the homing endonuclease I-*Ppo*I from the slime mould *Physarum polycephalum *[39], likewise *Porphyra fucicola *and *P. umbilicalis *HEG sequences [5]. As these sequences were not tested for endonuclease activity, they will be considered as HEG pseudogenes, as suggested by Müller *et al*. [5].

### *Cox*2-3 and *rbc*L-S regions analyses

*Cox*2-3 and *rbc*L-S regions were sequenced in addition to the SSU rDNA introns, to infer genealogical relationships of *PSA *populations. Divergence among *cox*2-3 haplotypes of the 40 *PSA *individuals sequenced ranged from 0.2% to 0.7%. These values are in accordance to the divergence found among *cox*2-3 haplotypes from *Grateloupia doryphora *(0.3% to 0.6%) from North Atlantic and North Pacific [19]. However, they differ significantly when compared to the divergence among *cox*2-3 region from *Batrachospermum helminthosum *individuals from North America, 0.3% to 6.5% [22] and from *Acanthophora spicifera *individuals from the Hawaiian Islands, where a single haplotype was observed [40].

*Rbc*L-S region has been employed in Rhodophyta as a marker at the inter-specific, intra-specific and intra-population levels [21,23,41-43], with levels of divergence of: 13% to18% for *Gracilaria *species [41] and 12.5% to 13.4% for individuals of the *Gymnogongrus *complex [42]. However, a unique haplotype from all the sampled range (ca. 800 km) was determined for the *rbc*L-S sequences from 25 *PSA *individuals.

### Population structure and genetic relationships of *PSA *populations

Genetic relationships of the 17 haplotypes (introns + HEG) obtained from *PSA *populations were accessed through network analyses. The network exhibit three main clusters suggesting a pattern of isolation by distance for the populations analyzed. The same grouping pattern was obtained for Fst analysis. Mantel's test corroborated the hypothesis indicated by the two previous analyses. Therefore, isolation by distance appears to be the basic process accounting for structure in *PSA *populations, manifested in a cline of HEG degeneration. Populations sampled at the southernmost end of the distribution present the entire His-Cys box motif, while the population sampled at the northernmost end of the distribution, considering the start codon proposed by Haugen *et al*. [4], has only eight amino acids of the HE. Distributed between these two extremes, are the intermediate-sized alleles.

The neutrality tests results for the intron without HEG indicated a fast population growth from an ancestor population with small effective number. At the same time, *cox*2-3 region results were marginally significant for the neutrality tests and *rbc*L-S region results showed no nucleotide variability. The low variability in these markers, also observed in the networks, is consistent with a demographic event of expansion from a population with low effective number affecting all loci. These results are not compatible to the HEG length polymorphism. The intron + HEG marker showed remarkable variation in length in the same individuals that presented few variations for the other markers. Therefore, in the same window of time, much more variation was accumulated in HEG than in sequence variation in the other three markers, which are probably under different selection constraints. Considering the recent population expansion for *PSA *along the Brazilian coast, degeneration of HEG was a very fast process. First, if we assume that a functional HEG have a cost to the host cell, then natural selection will increase the frequency of nonfunctional elements; and second, if we assume that the HEG was already fixed for *PSA *populations (there is no availability of insertion site), then the frequency of nonfunctional elements will increase due to low selection to keep a functional HEG [38].

Based on these assumptions, two different scenarios can be proposed to explain intron +HEG evolution in *PSA *populations: In the first scenario, the horizontal transfer of intron + HEG occurred in an ancestral individual, prior to the colonization of *PSA *populations in the Brazilian coast. Therefore, it is reasonable to believe that the same bottleneck event that was detected by the three markers (intron without HEG, *cox*2-3 and *rbc*L-S regions) probably had as a consequence the fixation in the population of the full length HEG (functional). If the largest introns are considered as the ancestral state, then the oldest populations are located in the southernmost end of the distribution, and as long as individuals migrate to the north, their HEGs tend to degenerate. This scenario is consistent with the proposed hypothesis for intron insertion and evolution in the order Bangiales based on phylogenetics analysis of SSU rDNA and respective group I introns – Intron horizontal transfer to an ancestor of the order Bangiales, followed by vertical inheritance and evolution within the order as proposed by Muller *et al *[5].

In the second scenario, the ancestral *PSA *individual lack intron + HEG. It is possible to suggest that the horizontal transfer of intron + HEG in *PSA *SSU rDNA occurred after the event of demographic expansion. In this case, the intron with the most degenerated HEG – with more deletions accumulated – is present in the oldest population. This state was found in the northernmost population, suggesting a horizontal transfer event to have occurred in an individual from the *PSA*-V population. After the horizontal transfer event, the functional HEG invaded the other *PSA *populations by gene flow, being the largest HEG present in the more recently invaded populations, at the southernmost end of distribution. Within the context of intron evolution in the order Bangiales, this scenario is possible assuming the hypothesis that more than one intron insertion events has occurred during the divergence of the order [5].

This is the first report addressing intron evolution focusing on only one species. Understanding the mechanisms beyond intron + HEG evolution has been a challenge, despite all the knowledge obtained for these elements.

### Self splicing assays

As self-splicing catalytic properties of group I introns are highly dependent on intron three-dimensional structure [44], we verified whether the occurrence of insertions in P1 paired element alters intron catalytic activities, checking if introns sizes variants self-splice *in vitro*. One way to check for intron excision is the confirmation of exons ligation [45].

The four intron size variants analyzed in this work self-spliced *in vitro*. Therefore, the occurrence of different size polymorphisms in intron P1 paired element do not refrain intron self-splicing mechanism, although there is a hypothesis that the presence of HEG may diminish self-splicing efficiency [1]. The loops are strategic localities for HEG insertion. HEG have been considered invasive mobile elements that remain neutral to the host when inserted into introns, becoming invisible to negative selection [6]. If group I introns lose their self-splicing capability due to the presence of a HEG, they both would probably be eliminated from the gene they were inserted, since there is a strong selection against non-functional rDNA genes.

## Conclusion

Commercial exploitation, mariculture and introduction of invasive species have been a major problem in the assignment of *Porphyra *geographic origins. Phenotypic plasticity along with a simple morphology is also an obstacle in *Porphyra *identification. Furthermore, scientific researches are more focused on taxonomy and phylogeny of the group than in population surveys. Population structure of *Porphyra spiralis *var. *amplifolia *could be assigned by HEG degeneration, although not by *cox*2-3 and *rbc*L-S regions. Therefore, intron size polymorphism is a suitable population marker for this species, and it can be rapidly detected using PCR assay.

The intron size polymorphism found in the PSA populations, corroborate the HEG cycle proposed by Goddard & Burt [38], indicating that the degenerated HEGs are reminiscent of the presence of a full-length and functional HEG, once fixed for *PSA *populations. The cline of HEG degeneration detected for *PSA *populations along the Southern Brazilian coast, determined the pattern of isolation by distance. Analyses with the other markers indicated a demographic event of expansion for *PSA*, from a population with low effective number. The maintenance of the HEG apparently does not refrain the ability of the intron to self-splice even when the different degrees of degeneration of these elements are present.

## Authors' contributions

DM carried out the research, carried out and interpreted the analyses and wrote the manuscript. MCO conceived the project, interpreted the analysis, supervised the project and revised the manuscript. FMM carried out and interpreted the population genetics analyses and wrote the manuscript. SRM co-supervised of the project and revised the manuscript. All authors participated in the discussions and approved the final manuscript.

## Supplementary Material

Additional file 1 **Alignment of the haplotypes (H1 to H17, Table **3**) of *Porphyra spiralis *var. *amplifolia *intron sequences**. Exons nucleotides are represented in lowercase letters, and intron and homing endonuclease gene nucleotides are represented in uppercase letters. Homing endonuclease open reading frame (ORF) start (484) and stop (28) positions are shadowed in gray. The orientation of the ORF is indicated by a horizontal arrow. Conserved nucleotides that forms the u*G pair are in positions 6 and 496, respectively. Line above sequences indicates Hys-Cys Box motif. Dashes in the alignment represent gaps. Click here for file

Additional file 2 ***In vitro *self-splicing reaction visualized in 0.7% agarose gel**. Lane 1, RNA ladder (Invitrogen); lane 2, *Porphyra spiralis *var. *amplifolia *(*PSA*)-V3; lane 3,*PSA*-G4; lane 4, *PSA*-T10; lane 5, *PSA*-L8. Expected sizes for each step of intron self-splicing are given in the right side of the figure. The largest bands are the product of intron cyclization [47]. Click here for file

Additional file 3 **Amplification of the cDNA confirming the ligation of the exons**. Lane 1, 100 bp DNA ladder (Promega); lanes 2 and 3,*Porphyra spiralis *var. *amplifolia *(*PSA*)-V3; lanes 4 and 5,*PSA*-G4; lanes 6 and 7, *PSA*-T10; lanes 8 and 9, *PSA*-L8. Click here for file
