# Supplementary material for: Group I introns and associated homing endonuclease genes reveals a clinal structure for Porphyra spiralis var. amplifolia (Bangiales, Rhodophyta) along the Eastern coast of South America
Source: BMC Evol Biol. 2008 Nov 7;8:308. doi: 10.1186/1471-2148-8-308 (PMC2585584; doi:10.1186/1471-2148-8-308)
Supplement: Additional file 1 — Alignment of the haplotypes (H1 to H17, Table 3) of Porphyra spiralis var. amplifolia intron sequences. Exons nucleotides are represented in lowercase letters, and intron and homing endonuclease gene nucleotides are represented in uppercase letters. Homing endonuclease open reading frame (ORF) start (484) and stop (28) positions are shadowed in gray. The orientation of the ORF is indicated by a horizontal arrow. Conserved nucleotides that forms the u*G pair are in positions 6 and 496, respectively. Line above sequences indicates Hys-Cys Box motif. Dashes in the alignment represent gaps. [file 1471-2148-8-308-S1.pdf]

```
              10        20        30        40        50        60        70        80        90
     ....|....|....|....|....|....|....|....|....|....|....|....|....|....|....|....|....|....|
H1   caagguUUCCGAAAGGGGCAAAGAGGACUAGUGACUCAUCCAAAGACUUACGAGAUGCUUUAUUAGACGCACUGCGGUGUAUGAAGACAC
H2   caagguUUCCGAAAGGGGCAAAAAGGACUAGUGACUCAUCCAAAGACUUACGAGAUGCUUUAUUACACGCACUGCGGUGUAUGAAGACAC
H3   caagguUUCCGAAAGGGGCAAAGAGGACUAGUGACUCAUCCAAAGACUUACGAGAUGCUUUAUUAGACGCACUGCG-UGUAUGAAGACAC
H4   caagguUUCCGAAAGGGGCAAAGAGGACUAGUGACUCAUCCAAAGACUUACGAGAUGCUUUAUUAGACGCACUGCGGUGUAUGAAGACAC
H5   caagguUUCCGAAAGGGGCAAAGAGGACUAGUGACUCAUCCAAAGACUUACGAGAUGCUUUAUUAGACGCACUGCGGUGUAUGAAGACAC
H6   caagguUUCCGAAAGGGGCAAAGAGGACUAGUGACUCAUCCAAAGACUUACGAGAUGCUUUAUUAGACGCACUGCG-UGUAUGAAGACAC
H7   caagguUUCCGAAAGGGGCAAAGAGGACUAGUGACUCAUCCAAAGACUUACGAGAUGCUUUAUUAGACGCACUGCGGUGUAUGAAGACAC
H8   caagguUUCCGAAAGGGGCAAAGAGGACUAGUGACUCAUCCAAAGACUUACGAGAUGCUUUAUUAG------------------------
H9   caagguUUCCGAAAGGGGCAAAGAGGACUAGUGACUCAUCCAAAGACUUACGAGAUGCUUUAUUAG------------------------
H10  caagguUUCCGAAAGGGGCAAAGAGGACUAGUGACUCAUCCAAAGACUUACGAGAUGCUUUAUUAG------------------------
H11  caagguUUCCGAAAGGGGCAAAGAGGACUAGUGACUCAUCCAAAGACUUACGAGAUGCUUUAUUAG------------------------
H12  caagguUUCCGAAAGGGGCAAAGAGGACUAGUGACUCAUCCAAAGACUUACGAGAUGCUUUAUUAG------------------------
H13  caagguUUCCGAAAGGGGCAAAGAGGACUAGUGACUCAUCCAAAGACUUACGAGAUGCUUUAUUAG------------------------
H14  caagguUUccgAAAGGGGCAAAGAGGACUAGUGACUCAUCCAAAGACUUACGAGAUGCUU-------------------------------
H15  caagguUUCCG-------------------------------------------------------------------------------
H16  caagguUUCCG-------------------------------------------------------------------------------
H17  caagguUUCCG-------------------------------------------------------------------------------

             100       110       120       130       140       150       160       170       180
     ....|....|....|....|....|....|....|....|....|....|....|....|....|....|....|....|....|....|
H1   UUG-AACUCCGGGUGGUUACGCAUGAGACGGCAGUAGAUGCGACUUUUGUUCAGGUCGCCGGACUCCAACGUUAAAUGCGCCUUGUUGAC
H2   UUG-AACUCCGGGUGGUUACGCAUGAGACGGCAGUAGAUGCGACUUUUGUUCAGGUCGCCGGACUCCAACGUUAAAUGCGCCUUGUUGAC
H3   UUGGAACUCCGGGUGGUUACGCAUGAGACGGCAGUAGAUGCGACUUUUGUUCAGGUCGCCGGACUCCAACGUUAAAUGCGCCUUGUUGAC
H4   UUG-AACUCCGGGUGGUUACGCAUGAGACGGCAGUAGAUGCGACUUUUGUUCAGGUCGCCGGACUCCAACGUUAAAUGCGCCUUGUUGAC
H5   UUG-AACUCCGGGUGGUUACGCAUGAGACGGCAGUAGAUGCGACUUUUGUUCAGGUCGCCGGACUCCAACGUUAAAUGCGCCUUGUUGAC
H6   UUG-AACUCCGGGUGGUUACGCAUGAGACGGCAGUAGAUGCGACUUUUGUUCAGGUCGCCGGACUCCAACGUUAAAUGCGCCUUGUUGAC
H7   UUG-AACUCCGGGUGGUUACGCAUGAGACGGCAGUAGAUGCGACUUUUGUUCAGGUCGCCGGACUCCAACGUUAAAUGCGCCUUGUUGAC
H8   ------------------------------------------------------------------------------------------
H9   ------------------------------------------------------------------------------------------
H10  ------------------------------------------------------------------------------------------
H11  ------------------------------------------------------------------------------------------
H12  ------------------------------------------------------------------------------------------
H13  ------------------------------------------------------------------------------------------
H14  ------------------------------------------------------------------------------------------
H15  ------------------------------------------------------------------------------------------
H16  ------------------------------------------------------------------------------------------
H17  ------------------------------------------------------------------------------------------

             190       200       210       220       230       240       250       260       270
     ....|....|....|....|....|....|....|....|....|....|....|....|....|....|....|....|....|....|
H1   ACACUUAGCGUUGUGGCACGUGUGGGACGCCUCUUCACCAGGGAGAGGGAGACGCUCAGUCGCUGCCACCGCGACAAUGAUGUGGCAAUA
H2   ACACUUAGCGUUGUGGCACGUGUGGGACGCCUCUUCACCAGGGAGAGGGAGACGCUCAGUCGCUGCCACCGCGACAAUGAUGUGGCAAUA
H3   ACACUUAGCGUUGUGGCACGUGUGGGACGCCUCUUCACCAGGGAGAGGGAGACGCUCAGUCGCUGCCACCGCGACAAUGAUGUGGCAAUA
H4   ACACUUAGCGUUGUGGCACGUGUGGGACGCCUCUUCACCAGGGAGAGGGAGACGCUCAGUCGCUGCCACCGCGACAAUGAUGUGGCAAUA
H5   ACACUUAGCGUUGUGGCACGUGUGGGACGCCUCUUCACCAGGGAGAGGGAGACGCUCAGUCGCUGCCACCGCGACAAUGAUGUGGCAAUA
H6   ACACUUAGCGUUGUGGCACGUGUGG-----------------------------------------------------------------
H7   ACACUUAGCGUUGUGGCACGUGUGG-----------------------------------------------------------------
H8   ------------------------------------------------------------------------------------------
H9   ------------------------------------------------------------------------------------------
H10  ------------------------------------------------------------------------------------------
H11  ------------------------------------------------------------------------------------------
H12  ------------------------------------------------------------------------------------------
H13  ------------------------------------------------------------------------------------------
H14  ------------------------------------------------------------------------------------------
H15  ------------------------------------------------------------------------------------------
H16  ------------------------------------------------------------------------------------------
H17  ------------------------------------------------------------------------------------------

             280       290       300       310       320       330       340       350       360
     ....|....|....|....|....|....|....|....|....|....|....|....|....|....|....|....|....|....|
H1   GUACUUCGUGCCUUGAUAGCGCAGUUGGACGUAACCGCCAGAGACCUUAGCA---GGGGCGUAGUCCGUCACAAUGCAGCCGUUCUCCAU
H2   GUACUUCGUGCCUUGAUAGCGCAGUUGGACGUAACCGCCAGAGACCUUAGCAGCAGGGGCGUAGUCCGUCACAAUGCAGCCGUUCUCCAU
H3   GUACUUCGUGCCUUGAUAGCGCAGUUGGACGUAACCGCCAGAGACCUUAGCA---GGGGCGUAGUCCGUCACAAUGCAGCCGUUCUCCAU
H4   GUACUUCGUGCCUUGAUAGCGCAGUUGGACGUAACCGCCAGAGACCUUAGCA---GGGGCGUAGUCCGUCACAAUGCAGCCGUUCUCCAU
H5   GUACUUCGUGCCUUGAUAGCGCAGUUGGACGUAACCGCCAGAGACCUUAGCA---GGGGCGUAGUCCGUCACAAUGCAGCCGUUCUCCAU
H6   -------------------------------------------------------------------------------------CCCAU
H7   -------------------------------------------------------------------------------------CCCAU
H8   ----------------------------------------------------------------UCCGUCACAAUGCAGCCGUUCUCCAU
H9   ----------------------------------------------------------------UCCGUCACAAUGCAGCCGUUCUCCAU
H10  ----------------------------------------------------------------UCCGUCACAAUGCAGCCGUUCUCCAU
H11  ----------------------------------------------------------------UCCGUCACAAUGCAGCCGUUCUCCAU
H12  ----------------------------------------------------------------UCCGUCACAAUGCAGCCGUUCUCCAU
H13  ----------------------------------------------------------------UCCGUCACAAUGCAGCCGUUCUCCAU
H14  ------------------------------------------------------------------------------------------
H15  ------------------------------------------------------------------------------------------
H16  ------------------------------------------------------------------------------------------
H17  ------------------------------------------------------------------------------------------
```

```
            370       380       390       400       410       420       430       440       450
    ....|....|....|....|....|....|....|....|....|....|....|....|....|....|....|....|....|....|
H1  GGAGUGCGAAUGCUUCUCUAACUGCUCCAGUACAGCUUGCUGGACGUGAUGGAGCAAGAACUUCUCGUAAGCG-UGGGUGGGCGCGACGU
H2  GGAGUGCGAAUGCUUCUCUAACUGCUCCAGUACAGCUUGCUGGACGUGAUGGAGCAAGAACUUCUCGUAAGCG-UGGGUGGGCGCGACGU
H3  GGAGUGCGAAUGCUUCUCUAACUGCUCCAGUACAGCUUGCUGGACGUGAUGGAGCAAGAACUUCUCGUAAGCG-UGGGUGGGCGCGACGU
H4  GGAGUGCGAAUGCUUCUCUAACUGCUCCAGUACAGCUUGCUGGACGUGAUGGAGCAAGAACUUCUCGUAAGCG-UGGGUGGGCGCGACGU
H5  GGAGUGCGAAUGCUUCUCUAACUGCUCCAGUACAGCUUGCUGGACGUGAUGGAGCAAGAACUUCUCGUAAGCG-UGGGUGGGCGCGACGU
H6  GGAGUGCGAAUGCUUCUCUAACUGCUCCAGUACAGCUUGCUGGACGUGAUGGAGCAAGAACUUCUCGUAAGCGGUGGGUGGGCGCGACGU
H7  GGAGUGCGAAUGCUUCUCUAACUGCUCCAGUACAGCUUGCUGGACGUGAUGGAGCAAGAACUUCUCGUAAGCGGUGGGUGGGCGCGACGU
H8  GGAGUGCGAAUGCUUCUCCAACUGCUCCAGUACAGCUUGCUGGACGUGAUGGAGCAAGAACUUCUCGUAAGCG-UGGGUGGGCGCGACGU
H9  GGAGUGCGAACGCUUCUCUAACUGCUCCAGUACAGCUUGCUGGACGUGAUGGAGCAAGAACUUCUCGUAAGCG-UGGGUGGGCGCGACGU
H10 GGAGUGCGAAUGCUUCUCUAACUGCUCCAGUACAGCUUGCUGGACGUGAUGGAGCAAGAACUUCUCGUAAGCG-UGGGUGGGCGCGACGU
H11 GGAGUGCGAAUGCUUCUCUAACUGCUCCAGUACAGCUUGCUGGACGUGAUGGAGCAAGAACUUCUCGUAAGCG-UGGUUGGGCGCGACGU
H12 GGAGUGCGAAUGCUUCUCUAACUGCUCCAGUACAGCUUGCUGGACGUGAUGGAGCAAGAACUUCUCGUAAGCG-UGGGUGGGCGCGACGU
H13 GGAGUGCGAAUGCUUCUCUAACUGCUCCAGUACAGCUUGCUGGACGUGAUGGAGCAAGAACUUCUCGUAAGCG-UGGGUGGGCGCGACGU
H14 ---------------CUCUAACUGCUCCAGUACAGCUUGCUGGACGUGAUGGAGCAAGAACUUCUCGUAAGCG-UGGGUGGGCGCGACGU
H15 ------------------------------------------------------------------------------------------
H16 ------------------------------------------------------------------------------------------
H17 ------------------------------------------------------------------------------------------

            460       470       480       490       500       510       520       530       540
    ....|....|....|....|....|....|....|....|....|....|....|....|....|....|....|....|....|....|
H1  CCAUGG-UAAUGAGACCUUCUCUGCAGGACACAUA-CCUACUGAAGCCUUUGCGGCCCGAAAGGGUGCGCGGUCACGACAUUAAAUAAAG
H2  CCAUGG-UAAUGAGACCUUCUCUGCAGGA--CAUACCCUACUGAAGCCUUUGCGGCCCGAAAGGGUGCGCGGUCACGACAUUAAAUAAAG
H3  CCAUGG-UAAUGAGACCUUCUCUGCAGGA--CAUACCCUACUGAAGCCUUUGCGGCCCGAAAGGGUGCGCGGUCACGACAUUAAAUAAAG
H4  CCAUGG-UAAUGAGACCUUCUCUGCAGGA--CAUA-CCUACUGAAGCCUUUGCGGCCCGAAAGGGUGCGCGGUCACGACAUUAAAUAAAG
H5  CCAUGG-UAAUGAGACCUUCUCUGCAGGA--CAUACCCUACUGAAGCCUUUGCGGCCCGAAAGGGUGCGCGGUCACGACAUUAAAUAAAG
H6  CCAUGG-UAAUGAGACCUUCUCUGCAUGA--CAUACCCUACUGAAGCCUUUGCGGCCCGAAAGGGUGCGCGGUCACGACAUUAAAUAAAG
H7  CCAUGG-UAAUGAGACCUUCUCUGCAGGA--CAUACCCUACUGAAGCCUUUGCGGCCCGAAAGGGUGCGCGGUCACGACAUUAAAUAAAG
H8  CCAUGG-UAAUGAGACCUUCUCUGCAGGA--CAUACCCUACUGAAGCCUUUGCGGCCCGAAAGGGUGCGCGGUCACGACAUUAAAUAAAG
H9  CCAUGGGUAAUGAGACCUUCUCUGCAGGA--CAUACCCUACUGAAGCCUUUGCGGCCCGAAAGGGUGCGCGGUCACGACAUUAAAUAAAG
H10 CCAUGGGUAAUGAGACCUUCUCUGCAGGA--CAUACCCUACUGAAGCCUUUGCGGCCCGAAAGGGUGCGCGGUCACGACAUUAAAUAAAG
H11 CCAUGGGUAAUGAGACCUUCUCUGCAGGA--CAUACCCUACUGAAGCCUUUGCGGCCCGAAAGGGUGCGCGGUCACGACAUUAAAUAAAG
H12 CCAUGGGUAAUGAGACCUUCUCUGCAGGA--CAUACCCUACUGAAGCCUUUGCGGCCCGAAAGGGUGCGCGGUCACGACAUUAAAUAAAG
H13 CCAUGG-UAAUGAGACCUUCUCUGCAGGA--CAUACCCUACUGAAGCCUUUGCGGCCCGAAAGGGUGCGCGGUCACGACAUUAAAUAAAG
H14 CCAUGG-UAAUGAGACCUUCUCUGCAGGA--CAUACCCUACUGAAGCCUUUGCGGCCCGAAAGGGUGCGCGGUCACGACAUUAAAUAAAG
H15 -----G-UAAUGAGACCUUCUCUGCAGGA--CAUACCCUACUGAAGCCUUUGCGGCCCGAAAGGGUGCGCGGUCACGACAUUAAAUAAAG
H16 -----G-UAAUGAGACCUUCUCUGCAGGA--CAUACCCUACUGAAGCCUUUGCGGCCCGAAAGGGUGCACGGUCACGACAUUAAAUAAAG
H17 -----G-UAAUGAGACCUUCUCUGCAGGA--CAUACCCUACUGAAGCCUUUGCGGCCCGAAAGGGUGCGCGGUCACGACAUUAAAUAAAG

            550       560       570       580       590       600       610       620       630
    ....|....|....|....|....|....|....|....|....|....|....|....|....|....|....|....|....|....|
H1  AGCCGUGUUCAACGCAAGUGACGGGG-AGAGAGGACUGAAAGUGACGGGGAAAAGUCGCUUUUGUUGGCGACUUUCCCGAGUGGGGGAUG
H2  AGCCGUGUUCAACGCAAGUGACGGGG-AGAGAGGACUGAAAGUGACGGGGAAAAGUCGCUUUUGUUGGCGACUUUCCCGAGUGGGGGAUG
H3  AGCCGUGUUCAACGCAAGUGACGGGG-AGAGAGGACUGAAAGUGACGGGGAAAAGUCGCUUUUGUUGGCGACUUUCCCGAGUGGGGGAUG
H4  AGCCGUGUUCAACGCAAGUGACGGGG-AGAGAGGACUGAAAGUGACGGGGAAAAGUCGCUUUUGUUGGCGACUUUCCCGAGUGGGGGAUG
H5  AGCCGUGUUCAACGCAAGUGACGGGG-AGAGAGGACUGAAAGUGACGGGGAAAAGUCGCUUUUGUUGGCGACUUUCCCGAGUGGGGGAUG
H6  AGCCGUGUUCAACGCAAGUGACGGGGGAGAGAGGACUGAAAGUGACGGGGAAAAGUCGCUUUUGUUGGCGACUUUCCCGAGUGGGGGAUG
H7  AGCCGUGUUCAACGCAAGUGACGGGG-AGAGAGGACUGAAAGUGACGGGGAAAAGUCGCUUUUGUUGGCGACUUUCCCGAGUGGGGGAUG
H8  AGCCGUGUUCAACGCAAGUGACGGGG-AGAGAGGACUGAAAGUGACGGGGAAAAGUCGCUUUUGUUGGCGACUUUCCCGAGUGGGGGAUG
H9  AGCCGUGUUCAACGCAAGUGACGGGG-AGAGAGGACUGAAAGUGACGGGGAAAAGUCGCUUUUGUUGGCGACUUUCCCGAGUGGGGGAUG
H10 AGCCGUGUUCAACGCAAGUGACGGGG-AGAGAGGACUGAAAGUGACGGGGAAAAGUCGCUUUUGUUGGCGACUUUCCCGAGUGGGGGAUG
H11 AGCCGUGUUCAACGCAAGUGACGGGG-AGAGAGGACUGAAAGUGACGGGGAAAAGUCGCUUUUGUUGGCGACUUUCCCGAGUGGGGGAUG
H12 AGCCGUGUUCAACGCAAGUGACGGGG-AGAGAGGACUGAAAGUGACGGGGAAAAGUCGCUUUUGUUGGCGACUUUCCCGAGUGGGGGAUG
H13 AGCCGUGUUCAACGCAAGUGACGGGG-AGAGAGGACUGAAAGUGACGGGGAAAAGUCGCUUUUGUUGGCGACUUUCCCGAGUGGGGGAUG
H14 AGCCGUGUUCAACGCAAGUGACGGGG-AGAGAGGACUGAAAGUGACGGGGAAAAGUCGCUUUUGUUGGCGACUUUCCCGAGUGGGGGAUG
H15 AGCCGUGUUCAACGCAAGUGACGGGG-AGAGAGGACUGAAAGUGACGGGGAAAAGUCGCUUUUGUUGGCGACUUUCCCGAGUGGGGGAUG
H16 AGCCGUGUUCAACGCAAGUGACGGGG-AGAGAGGACUGAAAGUGACGGGGAAAAGUCGCUUUUGUUGGCGACUUUCCCGAGUGGGGGAUG
H17 AGCCGUGUUCAACGCAAGUGACGGGG-AGAGAGGACUGAAAGUGACGGGGAAAAGUCGCUUUUGUUGGCGACUUUCCCGAGUGGGGGAUG

            640       650       660       670       680       690       700       710       720
    ....|....|....|....|....|....|....|....|....|....|....|....|....|....|....|....|....|....|
H1  CCAAGGUAUUAUAUCGCGGUAUAUAUAUAUCCUACAAUAGACGACGCAGUCAGUCCGAAAACCCAGUUGCUACCUUUUCAAAUUGCGGGA
H2  CCAAGGUAUUAUAUCGCGGUAUAUAUAUAUCCUACAAUAGACGACGCAGUCAGUCCGAAAACCCAGUUGCUACCUUUUCAAAUUGCGGGA
H3  CCAAGGUAUUAUAUCGCGGUAUAUAUAUAUCCUACAAUAGACGACGCAGUCAGUCCGAAAACCCAGUUGCUACCUUUUCAAAUUGCGGGA
H4  CCAAGGUAUUAUAUCGCGGUAUAUAUAUAUCCUACAAUAGACGACGCAGUCAGUCCGAAAACCCAGUUGCUACCUUUUCAAAUUGCGGGA
H5  CCAAGGUAUUAUAUCGCGGUAUAUAUAUAUCCUACAAUAGACGACGCAGUCAGUCCGAAAACCCAGUUGCUACCUUUUCAAAUUGCGGGA
H6  CCAAGGUAUUAUAUCGCGGUAUAUAUAUAUCCUACAAUAGACGACGCAGUCAGUCCGAAAACCCAGUUGCUACCUUUUCAAAUUGCGGGA
H7  CCAAGGUAUUAUAUCGCGGUAUAUAUAUAUCCUACAAUAGACGACGCAGUCAGUCCGAAAACCCAGUUGCUACCUUUUCAAAUUGCGGGA
H8  CCAAGGUAUUAUAUCGCGGUAUAUAUAUAUCCUACAAUAGACGACGCAGUCAGUCCGAAAACCCAGUUGCUACCUUUUCAAAUUGCGGGA
H9  CCAAGGUAUUAUAUCGCGGUAUAUAUAUAUCCUACAAUAGACGACGCAGUCAGUCCGAAAACCCAGUUGCUACCUUUUCAAAUUGCGGGA
H10 CCAAGGUAUUAUAUCGCGGUAUAUAUAUAUCCUACAAUAGACGACGCAGUCAGUCCGAAAACCCAGUUGCUACCUUUUCAAAUUGCGGGA
H11 CCAAGGUAUUAUAUCGCGGUAUAUAUAUAUCCUACAAUAGACGACGCAGUCAGUCCGAAAACCCAGUUGCUACCUUUUCAAAUUGCGGGA
H12 CCAAGAUAUUAUAUCGCGGUAUAUAUAUAUCCUACAAUAGACGACGCAGUCAGUCCGAAAACCCAGUUGCUACCUUUUCAAAUUGCGGGA
H13 CCAAGGUAUUAUAUCGCGGUAUAUAUAUAUCCUACAAUAGACGACGCAGUCAGUCCGAAAACCCAGUUGCUACCUUUUCAAAUUGCGGGA
H14 CCAAGGUAUUAUAUCGCGGUAUAUAUAUAUCCCACAAUAGACGACGCAGUCAGUCCGAAAACCCAGUUGCUACCUUUUCAAAUUGCGGGA
H15 CCAAGGUAUUAUAUCGCGGUAUAUAUAUAUCCUACAAUAGACGACGCAGUCAGUCCGAAAACCCAGUUGCUACCUUUUCAAAUUGCGGGA
H16 CCAAGGUAUUAUAUCGCGGUAUAUAUAUAUCCUACAAUAGACGACGCAGUCAGUCCGAAAACCCAGUUGCUACCUUUUCAAAUUGCGGGA
H17 CCAAGGUAUUAUAUCGCGGUAUAUAUAUAUCCUACAAUAGACGACGCAGUCAGUCCGAAAACCCAGUUGCUACCUUUUCAAAUUGCGGGA

           730       740       750       760       770       780       790       800       810
```

```
     ....|....|....|....|....|....|....|....|....|....|....|....|....|....|....|....|....|....|
H1   AUCCCCUAAGAGCCCAUCACUACCGCGGCCGUUGUGGAAAACACUCGGUGCAGCAGGCGAGGUAGUUCCCCGCUGGAUGGUAAUAAGGUG
H2   AUCCCCUAAGAGCCCAUCACUACCGCGGCCGUUGUGGAAAACACUCGGUGCAGCAGGCGAGGUAGUUCCCCGCUGGAUGGUAAUAAGGUG
H3   AUCCCCUAAGAGCCCAUCACUACCGCGGCCGUUGUGGAAAACACUCGGUGCAGCAGGCGAGGUAGUUCCCCGCUGGAUGGUAAUAAGGUG
H4   AUCCCCUAAGAGCCCAUCACUACCGCGGCCGUUGUGGAAAACACUCGGUGCAGCAGGCGAGGUAGUUCCCCGCUGGAUGGUAAUAAGGUG
H5   AUCCCCUAAGAGCCCAUCACUACCGCGGCCGUUGUGGAAAACACUCGGUGCAGCAGGCGAGGUAGUUCCCCGCUGGAUGGUAAUAAGGUG
H6   AUCCCCUAAGAGCCCAUCACUACCGCGGCCGUUGUGGAAAACACUCGGUGCAGCAGGCGAGGUAGUUCCCCGCUGGAUGGUAAUAAGGUG
H7   AUCCCCUAAGAGCCCAUCACUACCGCGGCCGUUGUGGAAAACACUCGGUGCAGCAGGCGAGGUAGUUCCCCGCUGGAUGGUAAUAAGGUG
H8   AUCCCCUAAGAGCCCAUCACUACCGCGGCCGUUGUGGAAAACACUCGGUGCAGCAGGCGAGGUAGUUCCCCGCUGGAUGGUAAUAAGGUG
H9   AUCCCCUAAGAGCCCAUCACUACCGCGGCCGUUGUGGAAAACACUCGGUGCAGCAGGCGAGGUAGUUCCCCGCUGGAUGGUAAUAAGGUG
H10  AUCCCCUAAGAGCCCAUCACUACCGCGGCCGUUGUGGAAAACACUCGGUGCAGCAGGCGAGGUAGUUCCCCGCUGGAUGGUAAUAAGGUG
H11  AUCCCCUAAGAGCCCAUCACUACCGCGGCCGUUGUGGAAAACACUCGGUGCAGCAGGCGAGGUAGUUCCCCGCUGGAUGGUAAUAAGGUG
H12  AUCCCCUAAGAGCCCAUCACUACCGCGGCCGUUGUGGAAAACACUCGGUGCAGCAGGCGAGGUAGUUCCCCGCUGGAUGGUAAUAAGGUG
H13  AUCCCCUAAGAGCCCAUCACUACCGCGGCCGUUGUGGAAAACACUCGGUGCAGCAGGCGAGGUAGUUCCCCGCUGGAUGGUAAUAAGGUG
H14  AUCCCCUAAGAGCCCAUCACUACCGCGGCCGUUGUGGAAAACACUCGGUGCAGCAGGCGAGGUAGUUCCCCGCUGGAUGGUAAUAAGGUG
H15  AUCCCCUAAGAGCCCAUCACUACCGCGGCCGUUGUGGAGAACACUCGGUGCAGCAGGCGAGGUAGUUCCCCGCUGGAUGGUAAUAAGGUG
H16  AUCCCCUAAGAGCCCAUCACUACCGCGGCCGUUGUGGAAAACACUCGGUGCAGCAGGCGAGGUAGUUCCCCGCUGGAUGGUAAUAAGGUG
H17  AUCCCCUAAGAGCCCAUCACUACCGCGGCCGUUGUGGAAAACACUCGGUGCAGCAGGCGAGGUAGUUCCCCGCUGGAUGGUAAUAAGGUG

              820       830       840       850       860       870       880       890       900
     ....|....|....|....|....|....|....|....|....|....|....|....|....|....|....|....|....|....|
H1   AUGGGAUUGUCUCUAGAGACUUGCAAUGGGCAAUCCGCAGCCAAGUCCCUGUCAAAUGGGAAAGGUUCACAGACUGUAAGGAAAGGGGUG
H2   AUGGGAUUGUCUCUAGAGACUUGCAAUGGGCAAUCCGCAGCCAAGUCCCUGUCAAAUGGGAAAGGUUCACAGACUGUAAGGAAAGGGGUG
H3   AUGGGAUUGUCUCUAGAGACUUGCAAUGGGCAAUCCGCAGCCAAGUCCCUGUCAAAUGGGAAAGGUUCACAGACUGUAAGGAAAGGGGUG
H4   AUGGGAUUGUCUCUAGAGACUUGCAAUGGGCAAUCCGCAGCCAAGUCCCUGUCAAAUGGGAAAGGUUCACAGACUGUAAGGAAAGGGGUG
H5   AUGGGAUUGUCUCUAGAGACUUGCAAUGGGCAAUCCGCAGCCAAGUCCCUGUCAAAUGGGAAAGGUUCACAGACUGUAAGGAAAGGGGUG
H6   AUGGGAUUGUCUCUAGAGACUUGCAAUGGGCAAUCCGCAGCCAAGUCCCUGUCAAAUGGGAAAGGUUCACAGACUGUAAGGAAAGGGGUG
H7   AUGGGAUUGUCUCUAGAGACUUGCAAUGGGCAAUCCGCAGCCAAGUCCCUGUCAAAUGGGAAAGGUUCACAGACUGUAAGGAAAGGGGUG
H8   AUGGGAUUGUCUCUAGAGACUUGCAAUGGGCAAUCCGCAGCCAAGUCCCUGUCAAAUGGGAAAGGUUCACAGACUGUAAGGAAAGGGGUG
H9   AUGGGAUUGUCUCUAGAGACUUGCAAUGGGCAAUCCGCAGCCAAGUCCCUGUCAAAUGGGAAAGGUUCACAGACUGUAAGGAAAGGGGUG
H10  AUGGGAUUGUCUCUAGAGACUUGCAAUGGGCAAUCCGCAGCCAAGUCCCUGUCAAAUGGGAAAGGUUCACAGACUGUAAGGAAAGGGGUG
H11  AUGGGAUUGUCUCUAGAGACUUGCAAUGGGCAAUCCGCAGCCAAGUCCCUGUCAAAUGGGAAAGGUUCACAGACUGUAAGGAAAGGGGUG
H12  AUGGGAUUGUCUCUAGAGACUUGCAAUGGGCAAUCCGCAGCCAAGUCCCUGUCAAAUGGGAAAGGUUCACAGACUGUAAGGAAAGGGGUG
H13  AUGGGAUUGUCUCUAGAGACUUGCAAUGGGCAAUCCGCAGCCAAGUCCCUGUCAAAUGGGAAAGGUUCACAGACUGUAAGGAAAGGGGUG
H14  AUGGGAUUGUCUCUAGAGACUUGCAAUGGGCAAUCCGCAGCCAAGUCCCUGUCAAAUGGGAAAGGUUCACAGACUGUAAGGAAAGGGGUG
H15  AUGGGAUUGUCUCUAGAGACUUGCAAUGGGCAAUCCGCAGCCAAGUCCCUGUCAAAUGGGAAAGGUUCACAGACUGUAAGGAAAGGGGUG
H16  AUGGGAUUGUCUCUAGAGACUUGCAAUGGGCAAUCCGCAGCCAAGUCCCUGUCAAAUGGGAAAGGUUCACAGACUGUAAGGAAAGGGGUG
H17  AUGGGAUUGUCUCUAGAGACUUGCAAUGGGCAAUCCGCAGCCAAGUCCCUGUCAAAUGGGAAAGGUUCACAGACUGUAAGGAAAGGGGUG

              910       920       930       940       950       960       970       980       990
     ....|....|....|....|....|....|....|....|....|....|....|....|....|....|....|....|....|....|
H1   CCGCUUCCUAUUACGCGGCGCUUAAGAGACAGUCGGUCCCCUGCGAAAGCAGAGUCCCGAAUUGGAGGAAAAGGCCCAUUAAAAGUGGGC
H2   CCGCUUCCUAUUACGCGGCGCUUAAGAGACAGUCGGUCCCCUGCGAAAGCAGAGUCCCGAAUUGGAGGAAAAGGCCCAUUAAAAGUGGGC
H3   CCGCUUCCUAUUACGCGGCGCUUAAGAGACAGUCGGUCCCCUGCGAAAGCAGAGUCCCGAAUUGGAGGAAAAGGCCCAUUAAAAGUGGGC
H4   CCGCUUCCUAUUACGCGGCGCUUAAGAGACAGUCGGUCCCCUGCGAAAGCAGAGUCCCGAAUUGGAGGAAAAGGCCCAUUAAAAGUGGGC
H5   CCGCUUCCUAUUACGCGGCGCUUAAGAGACAGUCGGUCCCCUGCGAAAGCAGAGUCCCGAAUUGGAGGAAAAGGCCCAUUAAAAGUGGGC
H6   CCGCUUCCUAUUACGCGGCGCUUAAGAGACAGUCGGUCCCCUGCGAAAGCAGAGUCCCGAAUUGGAGGAAAAGGCCCAUUAAAAGUGGGC
H7   CCGCUUCCUAUUACGCGGCGCUUAAGAGACAGUCGGUCCCCUGCGAAAGCAGAGUCCCGAAUUGGAGGAAAAGGCCCAUUAAAAGUGGGC
H8   CCGCUUCCUAUUACGCGGCGCUUAAGAGACAGUCGGUCCCCUGCGAAAGCAGAGUCCCGAAUUGGAGGAAAAGGCCCAUUAAAAGUGGGC
H9   CCGCUUCCUAUUACGCGGCGCUUAAGAGACAGUCGGUCCCCUGCGAAAGCAGAGUCCCGAAUUGGAGGAAAAGGCCCAUUAAAAGUGGGC
H10  CCGCUUCCUAUUACGCGGCGCUUAAGAGACAGUCGGUCCCCUGCGAAAGCAGAGUCCCGAAUUGGAGGAAAAGGCCCAUUAAAAGUGGGC
H11  CCGCUUCCUAUUACGCGGCGCUUAAGAGACAGUCGGUCCCCUGCGAAAGCAGAGUCCCGAAUUGGAGGAAAAGGCCCAUUAAAAGUGGGC
H12  CCGCUUCCUAUUACGCGGCGCUUAAGAGACAGUCGGUCCCCUGCGAAAGCAGAGUCCCGAAUUGGAGGAAAAGGCCCAUUAAAAGUGGGC
H13  CCGCUUCCUAUUACGCGGCGCUUAAGAGACAGUCGGUCCCCUGCGAAAGCAGAGUCCCGAAUUGGAGGAAAAGGCCCAUUAAAAGUGGGC
H14  CCGCUUCCUAUUACGCGGCGCUUAAGAGACAGUCGGUCCCCUGCGAAAGCAGAGUCCCGAAUUGGAGGAAAAGGCCCAUUAAAAGUGGGC
H15  CCGCUUCCUAUUACGCGGCGCUUAAGAGACAGUCGGUCCCCUGCGAAAGCAGAGUCCCGAAUUGGAGGAAAAGGCCCAUUAAAAGUGGGC
H16  CCGCUUCCUAUUACGCGGCGCUUAAGAGACAGUCGGUCCCCUGCGAAAGCAGAGUCCCGAAUUGGAGGAAAAGGCCCAUGAAAAGUGGGC
H17  CCGCUUCCUAUUACGCGGCGCUUAAGAGACAGUCGGUCCCCUGCGAAAGCAGAGUCCCGAAUUGGAGGAAAAGGCCCAUUAAAAGUGGGC

            1000      1010      1020      1030      1040      1050      1060      1070
     ....|....|....|....|....|....|....|....|....|....|....|....|....|....|....|....|....|
H1   UUUGGAGAGCCUCGGGAGGCCAGAGAGGAAAGGCUCCGCGCCCUCCUCACUCGGCUGGGUUCAAGUCCUUUGACUUUAUGuuccg
H2   UUUGGAGAGCCUCGGGAGGCCAGAGAGGAAAGGCUCCGCGCCCUCCUCACUCGGCUGGGUUCAAGUCCUUUGACUUUAUGuuccg
H3   UUUGGAGAGCCUCGGGAGGCCAGAGAGGAAAGGCUCCGCGCCCUCCUCACUCGGCUGGGUUCAAGUCCUUUGACUUUAUGuuccg
H4   UUUGGAGAGCCUCGGGAGGCCAGAGAGGAAAGGCUCCGCGCCCUCCUCACUCGGCUGGGUUCAAGUCCUUUGACUUUAUGuuccg
H5   UUUGGAGAGCCUCGGGAGGCCAGAGAGGAAAGGCUCCGCGCCCUCCUCACUCGGCUGGGUUCAAGUCCUUUGACUUUAUGuuccg
H6   UUUGGAGAGCCUCGGGAGGCCAGAGAGGAAAGGCUCCGCGCCCUCCUCACUCGGCUGGGUUCAAGUCCUUUGACUUUAUGuuccg
H7   UUUGGAGAGCCUCGGGAGGCCAGAGAGGAAAGGCUCCGCGCCCUCCUCACUCGGCUGGGUUCAAGUCCUUUGACUUUAUGuuccg
H8   UUUGGAGAGCCUCGGGAGGCCAGAGAGGAAAGGCUCCGCGCCCUCCUCACUCGGCUGGGUUCAAGUCCUUUGACUUUAUGuuccg
H9   UUUGGAGAGCCUCGGGAGGCCAGAGAGGAAAGGCUCCGCGCCCUCCUCACUCGGCUGGGUUCAAGUCCUUUGACUUUAUGuuccg
H10  UUUGGAGAGCCUCGGGAGGCCAGAGAGGAAAGGCUCCGCGCCCUCCUCACUCGGCUGGGUUCAAGUCCUUUGACUUUAUGuuccg
H11  UUUGGAGAGCCUCGGGAGGCCAGAGAGGAAAGGCUCCGCGCCCUCCUCACUCGGCUGGGUUCAAGUCCUUUGACUUUAUGuuccg
H12  UUUGGAGAGCCUCGGGAGGCCAGAGAGGAAAGGCUCCGCGCCCUCCUCACUCGGCUGGGUUCAAGUCCUUUGACUUUAUGuuccg
H13  UUUGGAGAGCCUCGGGAGGCCAGAGAGGAAAGGCUCCGCGCCCUCCUCACUCGGCUGGGUUCAAGUCCUUUGACUUUAUGuuccg
H14  UUUGGAGAGCCUCGGGAGGCCAGAGAGGAAAGGCUCCGCGCCCUCCUCACUCGGCUGGGUUCAAGUCCUUUGACUUUAUGuuccg
H15  UUUGGAGAGCCUCGGGAGGCCAGAGAGGAAAGGCUCCGCGCCCUCCUCACUCGGCUGGGUUCAAGUCCUUUGACUUUAUGuuccg
H16  UUUGGAGAGCCUCGGGAGGCCAGAGAGGAAAGGCUCCGCGCCCUCCUCACUCGGCUGGGUUCAAGUCCUUUGACUUUAUGuuccg
H17  UUUGGAGAGCCUCGGGAGGCCAGAGAGGAAAGGCUCCGCGCCCUCCUCACUCGGCUGGGUUCAAGUCCUUUGACUUUAUGuuccg
```
