# Supplementary figures and images for: Group I introns and associated homing endonuclease genes reveals a clinal structure for Porphyra spiralis var. amplifolia (Bangiales, Rhodophyta) along the Eastern coast of South America
Source: BMC Evol Biol. 2008 Nov 7;8:308. doi: 10.1186/1471-2148-8-308 (PMC2585584; doi:10.1186/1471-2148-8-308)

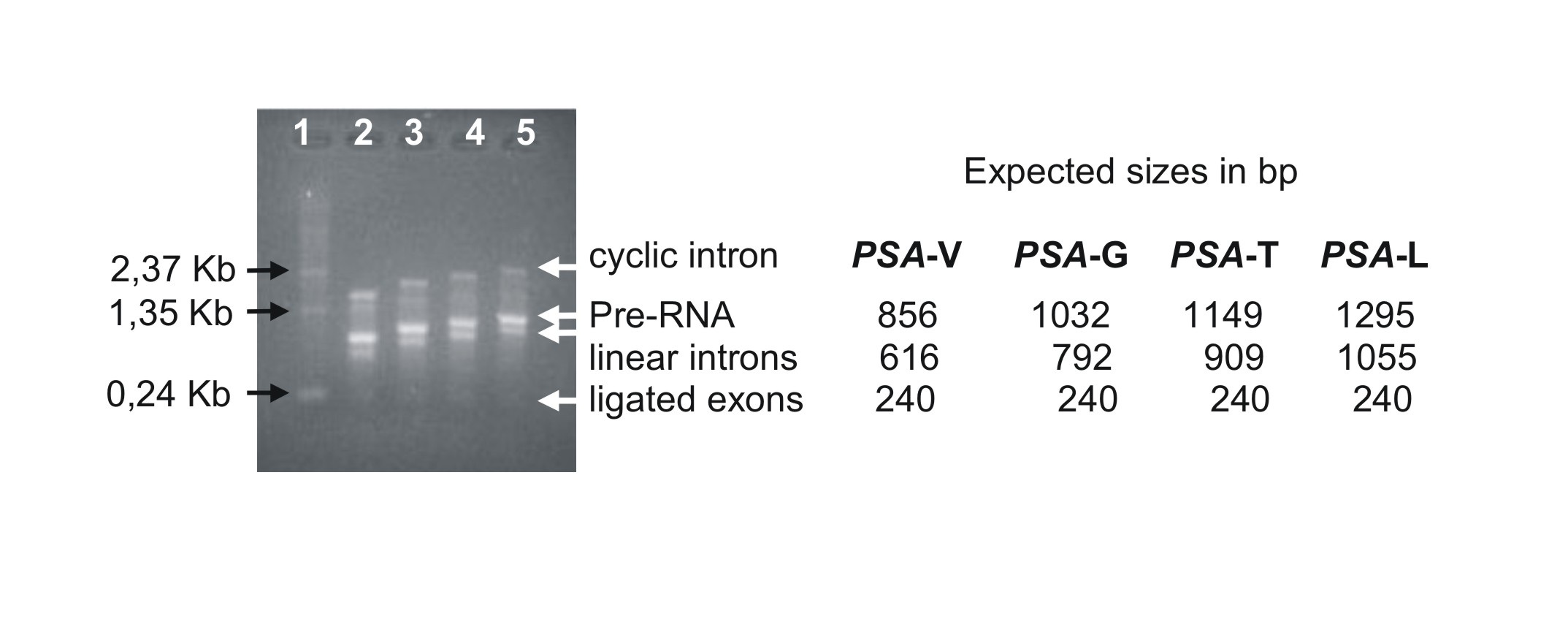

Supplement: Additional file 2 — In vitro self-splicing reaction visualized in 0.7% agarose gel. Lane 1, RNA ladder (Invitrogen); lane 2, Porphyra spiralis var. amplifolia (PSA)-V3; lane 3,PSA-G4; lane 4, PSA-T10; lane 5, PSA-L8. Expected sizes for each step of intron self-splicing are given in the right side of the figure. The largest bands are the product of intron cyclization [47]. [file 1471-2148-8-308-S2.jpeg]

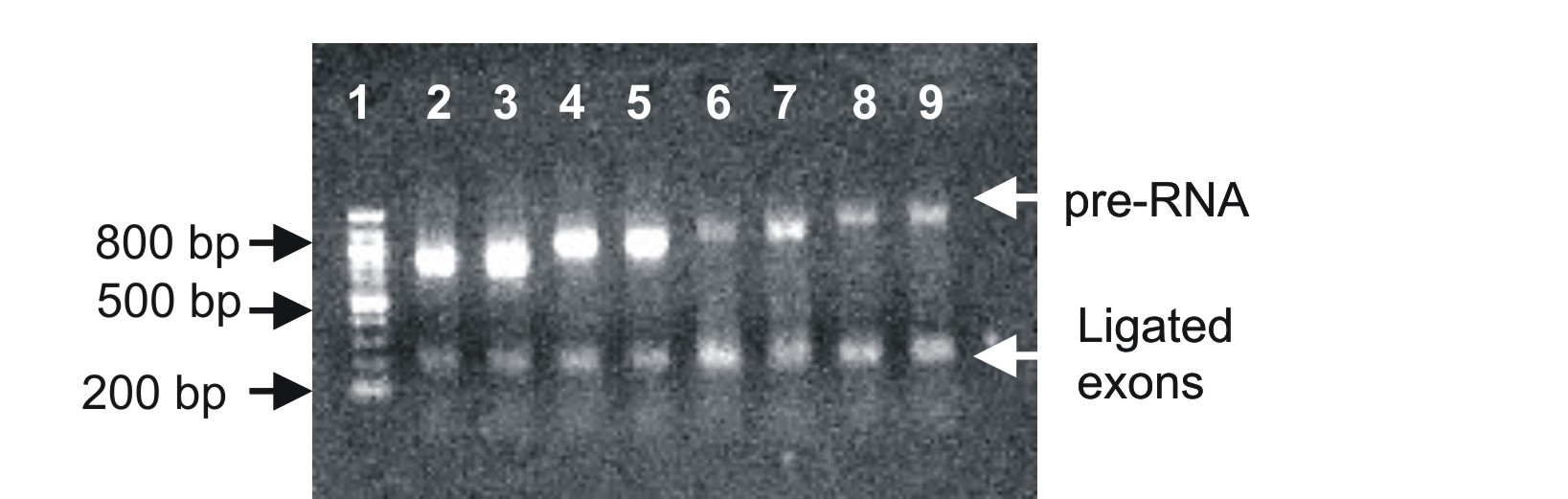

Supplement: Additional file 3 — Amplification of the cDNA confirming the ligation of the exons. Lane 1, 100 bp DNA ladder (Promega); lanes 2 and 3,Porphyra spiralis var. amplifolia (PSA)-V3; lanes 4 and 5,PSA-G4; lanes 6 and 7, PSA-T10; lanes 8 and 9, PSA-L8. [file 1471-2148-8-308-S3.jpeg]
